# The Effect of the Appropriate Timing of Radiotherapy on Survival Benefit in Patients with Metastatic Esophageal Cancer Who Have Undergone Resection of Primary Site: A SEER Database Analysis

**DOI:** 10.1155/2022/6086953

**Published:** 2022-03-16

**Authors:** Bingzhe Qiu, Yong Zhou, Ming Lou, Ke Zhang, Jiawei Lu, Jichun Tong

**Affiliations:** Department of Thoracic Surgery, The Affiliated Changzhou No. 2 People's Hospital of Nanjing Medical University, Changzhou, Jiangsu 213003, China

## Abstract

**Background:**

Metastatic esophageal cancer (MEC) is an advanced stage of esophageal cancer. However, still, resection of primary site and radiotherapy are considered treatment modalities to treat patients with MEC. Hence, this study is aimed at exploring the effect of the appropriate timing of radiotherapy on the survival benefit of these patients by comparing cancer-specific survival (CSS).

**Method:**

The patient information was obtained from the National Surveillance Epidemiology and End Results (SEER) database between the years 2004 and 2017. We used the SEER∗ STAT (V8.3.9.2) software to search and download data. Patients treated with pre- and postoperative radiotherapy were divided into two groups. The propensity score matching (PSM) analysis was performed to increase the comparability of data within two groups. We used the Kaplan-Meier method to analyze and compare the CSS between the two groups. The Cox risk model was used to analyze variables affecting patient survival.

**Results:**

A total of 599 patients with MEC who experienced resection of the primary site and radiotherapy were recruited. 144 pairings formed through PSM. The 5-year CSS was 23.0% and 11.7% for patients who have undergone pre- and postoperative radiotherapy, respectively. Patients who have undergone preoperative radiotherapy showed better CSS than those who received postoperative radiotherapy (*P* < 0.001). The multivariate Cox analysis of the entire cohort showed that age > 60 years at the time of diagnosis (HR = 1.481, 95% CI: 1.1341-1.934, and *P* = 0.04) and other histological types of esophageal cancer (HR = 1.581, 95% CI: 1.067-2.341, and *P* = 0.022) increased the risk of cancer-related death. Inversely, marriage (HR = 0.696, 95% CI: 0.514-0.942, and *P* = 0.019) and preoperative radiotherapy (HR = 0.664, 95% CI: 0.517-0.853, and *P* < 0.001) reduced the risk of death from cancer.

**Conclusions:**

For patients with MEC, preoperative radiotherapy might have a significant effect on the survival benefit over those who receive postoperative radiotherapy.

## 1. Introduction

Esophageal cancer is the sixth most common cancer and the seventh leading cause of cancer-related death worldwide [1]. Histologically, esophageal squamous cell carcinoma (ESCC) and esophageal adenocarcinoma (EAC) are the two main types of esophageal cancer [2]. In the last few decades, researchers have revealed the role of different treatment modalities of esophageal cancer and recognized radiotherapy as one of its important treatment options [3].

Despite a lot of scientific development, esophageal cancer still has a higher mortality rate because of being diagnosed at a later stage or the stage of metastasis [4]. Approximately 40% of patients with esophageal cancer are diagnosed at advanced stages, which significantly affects the prognosis [5]. The impact of preoperative chemoradiotherapy and adjuvant chemotherapy followed by resection in gastroesophageal cancer on relative survival rate has been studied by several researchers [6].

However, still, there is lacking evidence-based findings that could help to treat advanced esophageal cancer efficiently. Furthermore, the effect of the appropriate timing of radiotherapy in patients with MEC who have undergone primary site resection has not been well studied. Therefore, in this retrospective study, we aimed to find out the proper timing of radiotherapy and identify the risk factors affecting the prognosis in patients with MEC.

## 2. Materials and Methods

### 2.1. Patients Selection

Patients' information between the years 2004 and 2017 was collected from the SEER database. Patients with MEC who have undergone surgical resection of the primary site were included in this study.

Specifically, the inclusion criteria were as follows: (1) year of diagnosis between 2004 and 2017; (2) patients identified by “site record International Classification of Diseases for Oncology, Third Edition (ICD-0-3)/WHO 2008” with the term “Esophagus” as primary site; (3) patients with M1 esophageal cancer; (4) patients undergone surgical resection of the primary site; and (5) patients received radiotherapy either before or after surgery.

### 2.2. Factors and Groups

Variables that were taken into consideration in this study included the following: (1) age at diagnosis, (2) gender, (3) marital status, (4) race, (5) histologic type, (6) primary site-labeled, (7) grade, (8) AJCC T stage, (9) AJCC N stage, (10) tumor size, (11) survival months, and (12) vital status record.

**Figure 1:**
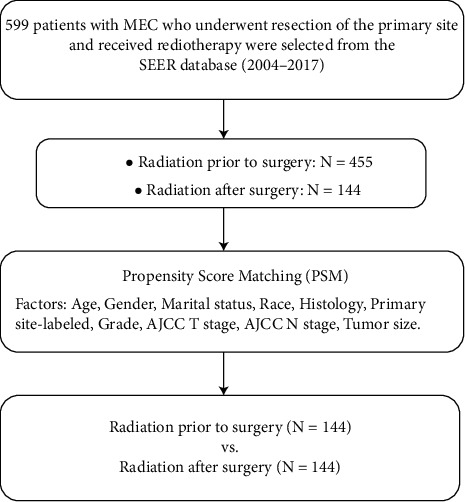
Flow chart of PSM.

To distribute the confounders evenly, minimize the inherent selection bias in the retrospective database, increase the similarity of clinical characteristics of patients, and enhance the comparability, the patients were divided into pre- and postoperative radiotherapy groups. The groups were made using PSM (1 : 1 matching ratio), based on variables 1-10 mentioned above. Details of patients' acquisition and grouping are shown in Figure 1.

### 2.3. Statistical Analysis

To perform PSM, R (version 3.4.4) for windows was used. A cross-tabulation was created to compare the clinical characteristics of the patients of the two groups. All the clinical characteristics were well balanced after PSM (all *P* > 0.05). The Kaplan-Meier method was used to analyze the survival curves, and the comparison between the two groups was done using the log-rank test. The differences between the two groups were considered statistically significant if the value of *P* < 0.05. Univariate analysis was performed using the Cox proportional risk model, and variables with *P* < 0.05 in the univariate analysis were included in the multivariate analysis to calculate the hazard ratio (HR). HR and corresponding 95% confidence interval (CI) were used to identify specific factors affecting CSS. The above statistical analyses were performed using SPSS (version 26.0).

## 3. Results

### 3.1. Patient Characteristics

A total of 599 patients with MEC who met all the inclusion and exclusion criteria were included in this study. Following PSM, the patients were divided into 144 pairs of preoperative and postoperative radiotherapy groups. The clinical characteristics of the patients in both groups were similar and significantly comparable as demonstrated in Table 1.

### 3.2. Survival Outcomes and Risk Factors

**Figure 2:**
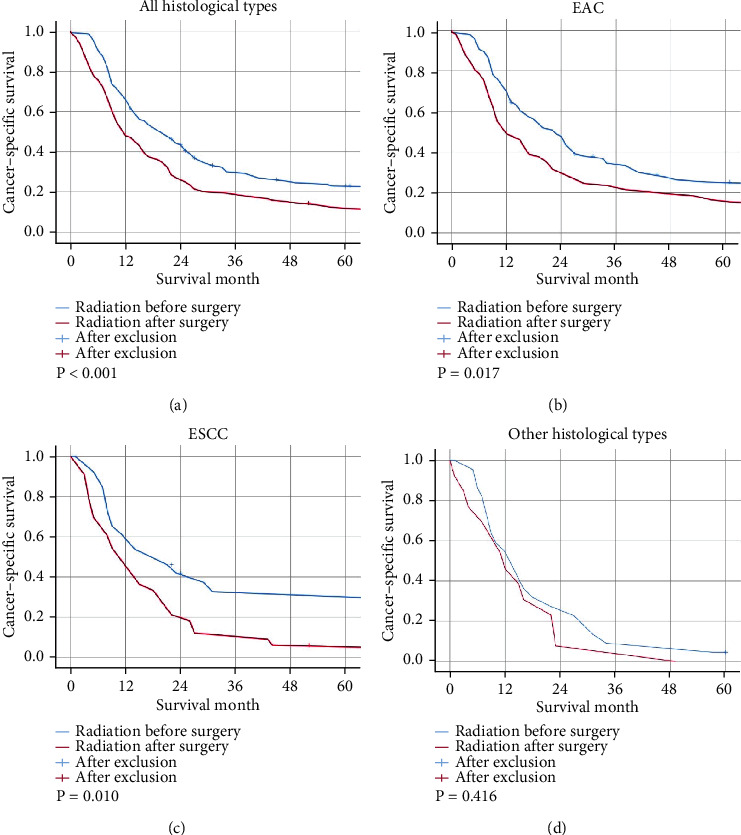
The Kaplan-Meier survival curves. (a) All histological types; (b) EAC; (c) ESCC; (d) other histological types.

The mean CSS of the patients with MEC who have undergone resection of the primary site after radiotherapy and before radiotherapy was 48 and 30 months, respectively. Furthermore, the 5-year CSS of the pre- and postoperative radiotherapy taking patients was estimated 23.0% and 11.7%, respectively. Kaplan-Meier survival curves also showed a significant CSS superiority with preoperative radiotherapy (log-rank test: *P* < 0.001; Figure 2(a)). The results for EAC (*P* = 0.017) and ESCC (*P* = 0.01), the major histological types of esophageal cancer, were also consistent (Figures 2(b) and 2(c)) with the previous outcomes. However, the sequence of radiotherapy did not show any significant effect on the survival benefit (*P* = 0.416) of the patients with other histological types of MEC who have undergone resection of the primary site as well (Figure 2(d)).

Since the sample size of patients in this study was not large, and the 5-year survival rate for patients with MEC was very low; we used Cox regression to analyze the individual factors affecting the 3-year survival rate among this type of patients. The results showed that the effects of the order of radiotherapy, age, marital status, histological type, primary site, grade, and AJCC N stage were statistically significant, and hence, we included them in the multifactorial analysis. The results of the multifactorial analysis showed that age more than 60 years at the time of diagnosis (HR = 1.481, 95% CI: 1.1341-1.934, and *P* = 0.04) and other histological types of esophageal cancer (HR = 1.581, 95% CI: 1.067-2.341) increased the risk of cancer mediated death. Conversely, marriage (HR = 0.696, 95% CI: 0.514-0.942, and *P* = 0.019) and preoperative radiotherapy (HR = 0.664, 95% CI: 0.517-0.853, and *P* < 0.001) reduced the risk of death from cancer (Table 2).

## 4. Discussion

Being a highly aggressive type of tumor, esophageal cancer is highly malignant and progresses rapidly. Surgical resection of the primary site is still considered the main treatment modality of esophageal cancer, and it can significantly improve the survival rate of patients if diagnosed at stages I-III [7]. Recently, neoadjuvant therapy has become one of the standard treatment options for patients with esophageal cancer [8]. Several studies have shown that the patients with locally advanced resettable esophageal cancer who have received surgery following preoperative neoadjuvant therapy have significantly improved relative survival rates with no significant adverse effects compared to those who have undergone surgery alone [9, 10]. Furthermore, the CROSS trial [11] suggested neoadjuvant therapy followed by surgery as the standard care for patients with resectable locally advanced esophageal cancer [12]. Though the role of different treatment modalities to treat locally advanced esophageal cancer has been extensively studied, the research on the therapeutic option to treat MEC remains inadequate and controversial. Hence, in this study, we attempted to investigate the effectiveness of surgery and the impact of the different timing (pre- or postoperative) of radiotherapy on the relative survival rate of the patient with MEC.

Recently, palliative care has been accepted as a standard method of care for patients with MEC. However, it has been proven less effective and with more adverse effects [13]. Therefore, there is an utmost necessity to explore more treatment strategies for MEC. Recent studies have reported that chemotherapy followed by surgery could help to prolong the relative survival rate in patients with MEC [14, 15]. Another study found that active primary radiotherapy could significantly improve the overall survival of patients with MEC [16]. Consequently, it is a new direction to explore whether the combination of radiotherapy and surgery can improve the length and/or relative rate of survival of the patients with MEC. A study by Van Daele et al. [17] showed that multimodality treatment including esophagectomy could be a treatment option to improve the long-term survival of patients with MEC. However, the sample size of their study was too small to comprehend the definitive effect of that surgery. In addition, Liu et al. [18] conducted a study in which 492 out of 5250 patients with MEC who have undergone primary resection showed significantly longer CSS. Outcomes of these studies demonstrate that surgery can benefit patients with MEC. However, none of them combined surgery with radiotherapy to provide new ideas to treat patients with MEC. A study by Xu et al. [19] reported that radiotherapy and palliative resection of the primary tumor could prolong the survival of patients with MEC. However, they have not conducted a definitive study on the appropriate sequence of radiotherapy and surgery. To minimize these limitations, in this study, we used a database with a larger population to investigate the appropriate timing of radiotherapy to improve the survival benefit of MEC patients who have undergone resection of the primary site. Our study has some success in obtaining statistically significant results as well.

However, this study still has some limitations. Firstly, it was retrospective and therefore subjected to unavoidable bias. Secondly, SEER provides information on the patient's surgical site, but not the detail of the procedure, the method, or the adverse effects followed by operation, nor the specific regimen of radiotherapy, which may lead to misinterpretation. Finally, in the two comparison groups after PSM, it has been interpreted that radiotherapy before surgery may shrink the tumor and change the stage; therefore, the treatment effect of the preoperative radiotherapy group was underestimated. This factor indicated that the survival benefit obtained with preoperative radiotherapy will be even greater than in our study. Despite these limitations, this study provided new evidence and ideas for the treatment of patients with MEC to improve their length of survival.

## 5. Conclusions

MEC is highly malignant and has a low survival rate. However, the age and marital status of the patients at the time of diagnosis could affect the survival of the patients negatively and positively, respectively. Based on our findings, we also recommend preoperative radiotherapy rather than postoperative radiotherapy for patients who are planning to undergo resection of the primary site.

## Figures and Tables

**Table 1:** Clinical characteristics and numbers of patients with MEC in two groups (preoperative radiotherapy and postoperative radiotherapy) after PSM.

Variable	Preoperative radiotherapy		Postoperative radiotherapy		Total	*P* valve
*Age*						
≤60	73	51.8%	68	48.2%	141	0.556
>60	71	48.3%	76	51.7%	147	
*Gender*						
Male	129	50.0%	129	50%	258	1
Female	15	50.0%	15	50%	30	
*Marital status*						
Unmarried	37	46.3%	43	53.8%	80	
Married	104	51.5%	98	48.5%	202	0.73
Unknown	3	50.0%	3	50%	6	
*Race*						
White	131	50.0%	131	50%	262	
Black	9	47.4%	10	52.6%	19	0.907
Others	4	57.1%	3	42.9%	7	
*Histology*						
Adenocarcinoma	96	49.5%	98	50.5%	194	
Squamous cell carcinoma	26	44.1%	33	55.9%	59	0.205
Others	22	62.9%	13	37.1%	35	
*Primary site*						
Upper	3	27.3%	8	72.7%	11	
Middle	19	47.5%	21	52.5%	40	0.422
Lower	115	52.0%	106	48%	221	
Overlapping	4	57.1%	3	42.9%	7	
Esophagus	3	33.3%	6	66.7%	9	
*Grade*						
I	3	50.0%	3	50%	6	
II	37	45.7%	44	54.3%	81	
III	84	52.8%	75	47.2%	159	0.864
IV	3	42.9%	4	57.1%	7	
Unknown	17	48.6%	18	51.4%	35	
*AJCC T*						
T0-T2	21	44.7%	26	55.3%	47	
T3-T4	108	52.4%	98	47.6%	206	0.421
TX	15	42.9%	20	57.1%	35	
*AJCC N*						
N0	40	48.2%	43	51.8%	83	
N+	103	51.5%	97	48.5%	200	0.352
Unknown	1	20.0%	4	80%	5	
*Tumor size*						
<5 cm	53	48.2%	57	51.8%	110	
≥5 cm	57	53.3%	50	46.7%	107	0.694
Unknown	34	47.9%	37	52.1%	71	

**Table 2:** Analysis of factors affecting cancer-specific survival in patients with MEC.

Variable	Univariate analysis		Multivariate analysis	
	3-year CSS (%)	*P*	HR (95% CI)	*P* valve
*Radiation order*		<0.001		
After surgery	18.8		Ref	
Before surgery	30.1		0.664 (0.517-0.853)	<0.001
*Age*		0.004		
≤60	30.2		Ref	
>60	18.7		1.481 (1.134-1.934)	0.004
*Gender*		0.074		
Male	22.5		/	
Female	37.4			
*Marital status*		0.017		
Unmarried	23.0		Ref	
Married	25.7		0.696 (0.514-0.942)	0.019
Unknown	/		/	
*Race*		0.585		
White	24.3			
Black	21.1		/	
Others	/			
*Histology*		0.043		
Adenocarcinoma	28.4		Ref	
Squamous cell carcinoma	20.9		0.844 (0.558-1.275)	0.420
Others	8.6		1.581 (1.067-2.341)	0.022
*Primary site*		<0.001		
Upper	18.2		Ref	
Middle	7.5		1.691 (0.813-3.518)	0.160
Lower	28.7		0.864 (0.429-1.775)	0.690
Overlapping	/		2.669 (0.854-8.339)	0.091
Esophagus	11.1		1.196(0.459-3.118)	0.714
*Grade*		0.002		
I	/		Ref	
II	28.4		0.463 (0.189-1.134)	0.092
III	22.9.		0.528 (0.221-1.264)	0.152
IV	/		1.702 (0.522-5.553)	0.378
Unknown	27.6		/	
*AJCC T*		0.189		
T0-T2	25.1			
T3-T4	25.5		/	
TX	16.3			
*AJCC N*		<0.001		
N0	27.0		Ref	
N+	23.9		1.262 (0.947-1.681)	0.112
Unknown	/		/	
*Tumor size*		0.302		
<5 cm	25.0		/	
≥5 cm	26.3			
Unknown	20.7			

Ref: reference. *P* < 0.05: statistically significant.

## Data Availability

The underlying data supporting this research could be found in the SEER∗ STAT (V8.3.9.2) software program.
